# Correction: Zhang et al. Free and Transient Vibration Analysis of Sandwich Piezoelectric Laminated Beam with General Boundary Conditions. *Materials* 2026, *19*, 136

**DOI:** 10.3390/ma19050884

**Published:** 2026-02-27

**Authors:** Xiaoshuai Zhang, Wei Fu, Zixin Ning, Ningze Sun, Yang Li, Ziyuan Yang, Sen Jiu

**Affiliations:** 1 China Ordnance Industry Group Aviation Ammunition Research Institute Co., Ltd., China North Industries Group Corporation Limited, Harbin 150030, China; 15545593315@163.com; 2 College of Mechanical and Electrical Engineering, Harbin Engineering University, Harbin 150001, China; 13521178147@163.com (Z.N.); 18045623837@163.com (Y.L.); yangziyuan1108@163.com (Z.Y.); js16637718196@163.com (S.J.); 3 College of Mechanical & Energy Engineering, Beijing University of Technology, Beijing 100124, China; snz2022@emails.bjut.edu.cn

**Additional Affiliation**

In the original publication [1], there was an error regarding the affiliations of Xiaoshuai Zhang. In addition to the current affiliation, they should also be marked with affiliation 2.

**Citation Correction**

In the original publication [1], reference 25 was incorrectly attributed to (“Chu, Z.; Wang, R.; Tian, S.; Wang, C.; Wu, L.; Wu, Q.; Yu, G. Fabrication and failure mechanisms of ultralight all-CFRP sandwich cylinders under axial compression. *Compos. Struct.* **2024**, *345*, 118386.”). The citation has now been corrected as follows:

25. Tornabene, F.; Viscoti, M.; Dimitri, R. Effect of thermal and electric coupling on the multifield response of laminated shell structures employing higher-order theories. *Compos. Struct.* **2025**, *354*, 118801.

The authors state that the scientific conclusions are unaffected. This correction was approved by the Academic Editor. The original publication has also been updated.
